# Complete Genomic Characterization of *Plum bark necrosis stem pitting–associated virus* Infecting Sweet Cherry in China

**DOI:** 10.1128/genomeA.00413-16

**Published:** 2016-05-19

**Authors:** Jiawei Wang, Ying Zhai, Weizhen Liu, Dongzi Zhu, Hanu R. Pappu, Qingzhong Liu

**Affiliations:** a Key Laboratory for Fruit Biotechnology Breeding of Shandong Province, Shandong Institute of Pomology, Taian, Shandong, China; b Department of Plant Pathology, Washington State University, Pullman, Washington, USA; c Department of Crop and Soil Sciences, Washington State University, Pullman, Washington, USA

## Abstract

*Plum bark necrosis stem pitting–associated virus* (PBNSPaV) causes the plum bark necrosis stem pitting–associated disease. We obtained the complete genome of a PBNSPaV isolate (PBNSPaV-TA) using small RNA deep sequencing followed by overlapping RT-PCR. To our knowledge, this is the first report of a completed genome of PBNSPaV identified from cherry trees.

## GENOME ANNOUNCEMENT

*Plum bark necrosis stem pitting–associated virus* (PBNSPaV) is a member of the genus *Ampelovirus*, family *Closteroviridae*, and has been considered as the causal agent of the plum bark necrosis stem pitting–associated disease (1). The PBNSPaV RNA genome encodes seven major open reading frames (ORFs), which is the simplest genome organization within the genus *Ampelovirus* (1). In recent years, the deep-sequencing approach is being increasingly applied for the broad-spectrum detection and population genetic analysis of PBNSPaV (2).

In a survey carried out for viruses on virus-infected sweet cherry (*Prunus avium* L.) trees of the cultivar “Red Lamp” in Shandong Province, China, we found that the infection of PBNSPaV may contribute to the symptoms of long and crinkled leaves, minor fruit-set, and late fruit-ripening in the “Red Lamp” cultivar. We named this PBNSPaV isolate PBNSPaV-TA. The complete genome of PBNSPaV-TA from sweet cherry was determined using small-RNA deep sequencing followed by overlapping RT-PCR.

Total RNA was extracted from the PBNSPaV-infected tree. Construction of a small RNA library was done by using a TruSeq small RNA sample prep kit (Illumina, San Diego, CA, USA), followed by 6% polyacrylamide gel electrophoresis for selection of low molecular weight RNAs. The Illumina HiSeq 2000 platform was used for the small RNA sequencing. From the pool of clean reads, contigs were assembled using CLC Genomics Workbench version 7.5 (CLC Bio, Aarhus, Denmark). The complete genome sequence of PBNSPaV-TA was obtained using small RNA sequencing, followed by overlapping RT-PCR and 5′ and 3′ rapid amplification of cDNA ends.

The complete genome sequence of PBNSPaV-TA consisted of 14,213 nucleotides (nt) obtained through deep sequencing and overlapping RT-PCR. The PBNSPaV-TA genome includes the 5′ untranslated region (UTR) (301 nt, from 1 to 301), ORF1a (7,032 nt, from 302 to 7333), ORF1b (1,575 nt, from 7335 to 8909), ORF2 (174 nt, from 8884 to 9057), ORF3 (1,590 nt, from 9061 to 10650), ORF4 (1,641 nt, from 10637 to 12277), ORF5 (978 nt, from 12335 to 13312), ORF6 (672 nt, from 13309 to 13980), and 3′ UTR (233 nt, from 13981 to 14213). There is a putative +1 ribosomal frameshift in ORF1b. ORF1a encodes a large 260-kDa polyprotein, which consists of putative papain-like protease, methyltransferase, and helicase. ORF1b encodes a 64.1-kDa RNA-dependent RNA polymerase (RdRp). ORF2 encodes a 6.3-kDa hydrophobic protein. ORF3 encodes a 57.4-kDa heat shock protein homolog (HSP70h). ORFs 4, 5, and 6 encode a 61.6-kDa protein of unknown function, a 35.9-kDa capsid protein (CP), and a 25.2-kDa minor capsid protein (CPm), respectively (1). To our knowledge, this is the first report of a completed genome of PBNSPaV identified from cherry trees. Our research provides a platform for further investigation into interactions between the PBNSPaV genotype and the host phenotype of PBNSPaV-infected sweet cherry.

### Nucleotide sequence accession number.

The whole-genome sequence of PBNSPaV-TA has been deposited in GenBank under the accession number KU240013.
